# Oxidized Regenerated Cellulose versus Polyglycolic Acid for Pleural Coverage in Pneumothorax Surgery

**DOI:** 10.3390/jcm12113705

**Published:** 2023-05-27

**Authors:** Wongi Woo, Bong Jun Kim, Duk Hwan Moon, Du-young Kang, Sungsoo Lee, Tae Yun Oh

**Affiliations:** 1 Department of Thoracic and Cardiovascular Surgery, Gangnam Severance Hospital, Yonsei University College of Medicine, Seoul 06273, Republic of Korea; woopendo@gmail.com (W.W.);; 2 Department of Thoracic and Cardiovascular Surgery, National Health Insurance Service Ilsan Hospital, Goyang 10444, Republic of Korea; 3 Department of Thoracic and Cardiovascular Surgery, Kangbuk Samsung Hospital, Sungkyunkwan University College of Medicine, Seoul 03181, Republic of Korea; chtoh.oh@samsung.com

**Keywords:** pneumothorax, pleural coverage, oxidized regenerated cellulose, polyglycolic acid

## Abstract

**Objectives:** Although surgical intervention for spontaneous pneumothorax (SP) reduces the recurrence rate, thoracoscopic surgery is associated with greater postoperative recurrence rates than open thoracotomy. A polyglycolic acid (PGA) sheet or oxidized regenerated cellulose (ORC) mesh can therefore be used for additional coverage after thoracoscopic surgery, and this study compared the clinical impacts of these two materials. **Methods:** From 2018 to 2020, 262 thoracoscopic surgeries for primary SP were performed, of which 125 patients were enrolled in this study, and 48 and 77 patients received ORC and PGA coverage, respectively. The clinical characteristics and surgical procedures were reviewed, and the recurrence rates were compared. To obtain more comprehensive evidence, we performed a literature review and meta-analysis comparing ORC and PGA coverage. **Results:** There were no significant differences in patient characteristics between the two groups. Operating time was slightly shorter in the ORC group than in the PGA group (*p* = 0.008). The pneumothorax recurrence rate was similar in both groups (PGA: 10.4%, ORC: 6.2%, *p* = 0.529), but the recurrence-free interval was significantly longer (*p* = 0.036) in the ORC (262 days) than in the PGA (48.5 days) group. The literature review identified three relevant studies, and the meta-analysis revealed no difference in pneumothorax recurrence rate between the two coverage materials. **Conclusions:** The two visceral pleural coverage materials, PGA and ORC, did not show significant differences in postoperative pneumothorax recurrence. Therefore, if applied appropriately, the choice of material between ORC and PGA for thoracoscopic pneumothorax surgery does not have a significant impact on the clinical outcome.

## 1. Introduction

Spontaneous pneumothorax (SP) is a condition in which air is trapped within the pleural space, with or without trauma, and frequently recurs. The annual prevalence rate of SP in Korea is 39–66 per 100,000 individuals, which is slightly higher than reports in other countries [1,2,3,4]. There are various treatment options for SP, and common treatment strategies include oxygen therapy, chest drainage, and thoracoscopic surgery.

Surgical intervention for SP is indicated to both control the primary air leakage site in cases of persistent air leak through a chest drain, and reduce recurrence. Video-assisted thoracoscopic surgery (VATS) is more beneficial in terms of blood loss, postoperative pain, and hospital stay compared with open thoracotomy, and is therefore the preferred approach for pneumothorax surgery [5]. VATS is, however, associated with a high incidence of postoperative recurrence, which remains a concern [6].

To lower the recurrence risk, additional procedures, such as pleural coverage using a polyglycolic acid (PGA) sheet or an oxidized regenerated cellulose (ORC) mesh, or chemical/mechanical pleurodesis, have been performed after VATS bullectomy [7,8,9]. Visceral coverage using a PGA sheet was first reported by Mukaida et al. [10] to decrease postoperative recurrence via PGA-induced adhesion, a mechanism demonstrated in several animal studies [11]. However, pneumothorax recurrence after PGA coverage still occurs frequently. In such cases, surgeons experience difficulty during reoperation due to severe PGA-induced pleural adhesions. Visceral coverage with an ORC sheet was therefore introduced owing to its low risk of severe adhesion. ORC reinforces the visceral pleura with pleural thickening without adhesion formation via mesothelial–mesenchymal transition [12].

However, relatively few reports have compared the postoperative recurrence rates of PGA sheet and ORC mesh coverage. This study therefore aimed to compare ORC and PGA in terms of early surgical outcomes and postoperative pneumothorax recurrence rates. Additionally, we reviewed the current literature to comprehensively evaluate whether ORC or PGA is appropriate for pneumothorax surgery.

## 2. Subjects

VATS procedures were performed on 115 patients with pneumothoraxes at Hospital A from 2018 to 2020. Among these patients, 48 underwent surgery for primary spontaneous pneumothorax (PSP) and received ORC mesh coverage (ORC group) after bullectomy. Among them, there were no patients who had no coverage or PGA sheets. During the same period, 214 VATS procedures for pneumothorax were performed for PSP in hospital B, and 77 patients received PGA sheet coverage (PGA group) after the same procedure. Patients with no coverage (*n* = 6) and double coverage (PGA + ORC, *n* = 131) were excluded from the analysis. All patients underwent chest computed tomography (CT) before surgery, and their electronic medical records, including clinical characteristics and surgical procedures, were reviewed.

## 3. Methods

This study was approved by the institutional review board of the two institutions (IRB No. 3-2021-0491). The requirement for informed consent from individual patients was waived owing to the retrospective design of the study.

### 3.1. VATS Procedure

**Figure 1:**
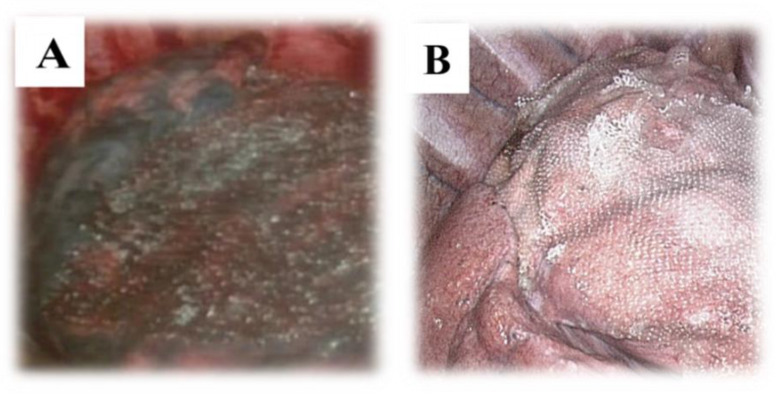
Thoracoscopic view of the two coverage materials: (**A**) PGA, (**B**) ORC. ORC—oxidized regenerated cellulose; PGA—polyglycolic acid.

General anesthesia was induced per the standard protocol, and patients were intubated with a single- or double-lumen endotracheal tube. Patients were placed in the lateral decubitus position. As previously reported, three-port VATS using two 5 mm thoracoscopic trocars and one 12 mm trocar was performed [10]. A 5 mm port was used to introduce a 5 mm, 30-degree thoracoscope and VATS instruments, such as a 5 mm grasper, and a 12 mm trocar was used for the other VATS instruments, the endoscopic stapler, and the removal of specimens. In hospital B, 20 of the 78 patients (25.6%) underwent single-port VATS (12-mm). To secure a surgical field of view in patients with single-lumen endotracheal intubation, VATS bullectomy was performed under CO_2_ insufflation using check-valved trocars, and bullae were resected using an endoscopic stapler. In the PGA group, the stapler line was covered with an absorbable PGA sheet (Neoveil, Gunze Ltd., Kyoto, Japan) (Figure 1A). In the ORC group, more than one lung-segmental surface, including the stapler line, was covered with an ORC mesh (Ethicon SURGICEL^®^ absorbable Hemostat gauze, Johnson & Johnson, Brunswick, NJ, USA) (Figure 1B). A 20 Fr chest tube was placed through the inferior 5 mm trocar. To affix the coverage material, fibrin sealant (Beriplast^®^ P, Aventis Behring, Marburg, Germany) was applied in the PGA group, and autologous blood was used in the ORC group. After confirming sufficient lung expansion and stable attachment of the covering materials to the lung surface through the thoracoscope, the other trocar sites were closed.

### 3.2. Postoperative Course

In both institutions, patients were subjected to −15 cmH_2_O suction using a chest bottle system immediately after the operation to enhance pleural symphysis. Chest radiography (CXR) was performed daily to confirm full lung expansion. Postoperative air leaks were qualitatively assessed by asking patients to cough while observing the water column and seal for air bubbles. In the absence of air leaks, the chest tube was removed on the second postoperative day. In cases of air leaks lasting more than 3 days (hospital B) or 1 week (hospital A), pleurodesis was performed using Mistletoe extract (Abnoba Viscum F; ABNOBA Helmittel GmbH, Pforzheim, Germany) or 50% glucose solution. In the absence of air space enlargement on CXR, patients were discharged the day after chest tube removal. Patients were followed up non-urgently 2 weeks post-discharge to assess the wound and any CXR abnormalities. Postoperative recurrence was defined as any ipsilateral recurrent pneumothorax after the resolution or full expansion of the operated lung.

### 3.3. Literature Review

A PubMed database search for articles published in English from inception until 22 June 2022 using the medical terms ((pneumothorax) AND ((regenerated cellulose) OR (Surgicel)) AND (polyglycolic acid)) yielded four studies. One of these studies was excluded as it compared double coverage (ORC + PGA) with ORC coverage. The other three studies [8,13,14] included 102 patients in the ORC group and 470 patients in the PGA group. We compared the recurrence rates in these studies, as well as the present study, according to the two coverage materials.

### 3.4. Statistical Analysis

The continuous variables are presented as medians and interquartile ranges since they all exhibited non-normal distributions, and were compared using the Mann–Whitney U test. Fisher’s exact test was used to compare categorical variables of the ORC and PGA groups. 

In the meta-analysis comparing the two coverage materials, recurrence risk was expressed as the relative risk (RR) and the 95% confidence interval (CI). A random-effects model was used to demonstrate the comparison as significant heterogeneity, which was expressed as I^2^ > 50%, among the included studies. Publication bias was evaluated using a funnel plot. 

All statistical analyses were performed with SPSS version 24.0 (IBM Corp., Armonk, NY, USA) and Review Manager (RevMan) software version 5.2.3 (The Nordic Cochrane Centre, Copenhagen, Denmark).

## 4. Results

The demographic and clinical characteristics of the 48 and 77 patients in the ORC and PGA groups are summarized in Table 1. In both groups, the median age was very young (20.0 (ORC) and 19.0 (PGA) years), most were male, and more than half of patients (62.5% (30/48) in ORC; 58.4% (45/77) in PGA) had a history of pneumothorax, although this did not differ significantly between the two groups. Indications for surgery included prolonged air leak, the presence of bullae on a CT (size over 5 mm), or recurrent pneumothorax. Among patients with recurrent pneumothorax, they were classified as having prolonged air leak or the presence of bullae in surgical indication if they had these conditions. There were no significant differences in the demographics and pneumothorax-related characteristics between the two coverage-material groups.

The clinical outcomes after surgery are summarized in Table 2. Although the chest tube indwelling time was similar for the two groups, the operating time was significantly longer in the PGA group (*p* = 0.008). Postoperative pleurodesis was performed in only four (5.2%) patients in the PGA group. There was no difference in the postoperative recurrence rate; however, the median recurrence-free interval was significantly longer in the ORC group (262 days) than in the PGA group (48.5 days) (*p* = 0.036). Treatment after recurrence was comparable between the two groups, and approximately one-third of the patients required additional surgery.

**Figure 2:**
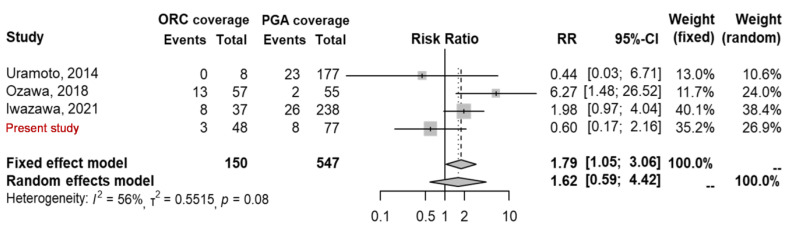
Forest plot comparing the recurrence rates of the two coverage materials [8,13,14]. ORC—oxidized regenerated cellulose; PGA—polyglycolic acid.

**Figure 3:**
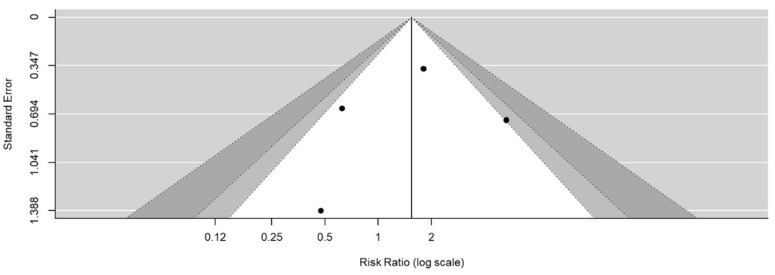
Funnel plot illustrating publication bias of the included studies.

A systematic review of three studies (Table 3) retrieved through a PubMed/Medline search, comparing the two coverage materials, was conducted. The follow-up period and the number of participants varied. A meta-analysis of postoperative recurrence, performed after including our study, found no significant difference between the two coverage materials (RR: 1.62, 95% CI: 0.59–4.42, *p* = 0.08, I^2^: 56%) (Figure 2). The funnel plot was balanced in terms of publication bias (Figure 3).

## 5. Discussion

Since the introduction of visceral pleural coverage methods for lowering the recurrence risk following VATS for pneumothorax, coverage material selection has challenged thoracic surgeons. Our study compared ORC and PGA coverage across two institutions and performed a meta-analysis incorporating related studies to evaluate current evidence regarding this issue. The results showed no definite preference for ORC or PGA in terms of postoperative recurrence, which suggests that postoperative recurrence is dependent on clinical factors rather than on the biological properties of coverage materials. 

Fujiwara et al. suggested that age (<30 years) is a risk factor for recurrence after adjusting for confounding factors including surgical strategies [15], and similar age-related factors were found to be related to recurrence in another study [16]. Furthermore, postoperative prolonged air leakage [17], an inability to identify bullae on a preoperative CT [18], and the surgeons’ experience [19] have been reported as recurrence risk factors. Surgical techniques, such as the number of ports in pneumothorax surgery, did not differ with regard to postoperative recurrence [20]. Although the benefit of staple line coverage has been proven in previous studies [21,22], if patients do not have significant risk factors for recurrence, the choice of coverage material may not lead to further improved clinical outcomes.

Further assessment is needed when interpreting differences in the recurrence-free interval between the two groups, which may be related to the histopathological mechanisms of the materials. There are various reasons for pneumothorax recurrence after VATS, including overlooked small bullae, newly developed bullae, and neo-bullae near the stapler line [23]. In this study, one segmental surface, in addition to the staple line, was covered in the ORC group. Therefore, a larger coverage area could help minimize the risk of early neo-bullae formation near the staple line. In addition, early recurrence in the PGA group may be related to the healing process or detachment of the coverage materials from the lung [24]. To understand different recurrence-free intervals, further studies including more patients with follow-up and sequential radiologic examination are needed.

The longer operating times in the PGA group are thought to be related to surgical procedures. In contrast to the ORC group, where every patient underwent VATS via three ports, a quarter of the patients in the PGA group underwent single-port VATS. Due to surgical intricacies of the single-port procedure [25], it could take longer than conventional three-port VATS; however, the 6 min difference does not seem to impact the general clinical course.

If there are no clinical benefits in terms of recurrence prevention, the pros and cons of each material should be considered when they are applied. The most distinguishing point is its impact on the formation of severe pleural adhesion. PGA is well known for its formation of dense pleural symphysis; it often increases the risk of bleeding and surgical time in the second thoracic surgery [26]. Compared to PGA, ORC dissolves more quickly and has a lesser foreign-body inflammatory reaction [27,28]. Based on this information, surgeons need to assess the potential benefits and risks to patients of each material and decide upon a suitable mesh. 

This study had several limitations. First, the interpretation of the meta-analysis needs to consider possible confounding factors of the included patients. Moreover, the accuracy of our findings is likely reduced, as the four studies, including our study, were retrospectively designed. Second, the area of pleural coverage of the two groups was not quantitatively compared, and a larger coverage area in ORC could lead to better results. Third, an additional sealant, such as autologous blood or fibrin glue, could influence the results. In the studies included in the meta-analysis, the information about this issue was also insufficient. 

## 6. Conclusions

Postoperative pneumothorax recurrence did not differ significantly between the two visceral pleural coverage materials (PGA and ORC). A meta-analysis based on the current literature also revealed that ORC is not inferior in terms of preventing recurrence. Therefore, the choice of material between ORC and PGA during thoracoscopic surgery for pneumothorax does not seem to have a significant impact on the clinical course.

## Figures and Tables

**Table 1:** Patients’ demographics and the clinical characteristics of pneumothorax.

	Coverage Method		
Variables	ORC	PGA	*p*-Value
	N = 48	N = 77	
Age, years	20.0 [17.8, 31.3]	19.0 [17.0, 26.0]	0.323
Gender			0.434
Female	5 (10.4)	13 (16.9)	
Male	43 (89.6)	64 (83.1)	
Previous pneumothorax history			0.710
Yes	30 (62.5)	45 (58.4)	
No	18 (37.5)	32 (41.6)	
Site of pneumothorax			0.742
Left	26 (54.2)	40 (51.9)	
Right	22 (45.8)	35 (45.5)	
Both	0 (0.0)	2 (2.6)	
Indication for surgery			0.224
Prolonged air leakage or the existence of bullae ^¶^	29 (60.4)	36 (46.8)	
Recurrent pneumothorax	19 (39.6)	41 (53.2)	
Lesion from bullae or air-leakage			0.914
RUL	19 (39.6)	31 (40.8)	
LUL	21 (43.8)	34 (44.7)	
RLL	2 (4.2)	1 (1.3)	
LLL	2 (4.2)	1 (1.3)	
RML	1 (2.1)	1 (1.3)	
LUL + LLL	3 (6.2)	6 (7.9)	
LUL + LLL + RUL	0 (0.0)	1 (1.3)	
RML + RLL	0 (0.0)	1 (1.3)	

Data are presented as n (%), n/N (%), or median (interquartile range). ORC—oxidized regenerated cellulose; PGA—polyglycolic acid; RUL—right upper lobe; RML—right middle lobe; RLL—right lower lobe; LUL—left upper lobe; LLL—left lower lobe. ^¶^ prolonged air leakage means air leakage over 2 days; the identification of bullae was performed via chest computed tomography evaluation.

**Table 2:** Clinical outcomes of patients according to the coverage methods.

	Coverage Method		
Variables	ORC	PGA	*p*-Value
	N = 48	N = 77	
Operation time, minutes	30.0 [25.0, 35.0]	36.0 [28.0, 43.0]	0.008
Chest tube indwelling time, days	2.0 (2.0, 2.0)	2.0 (2.0, 2.0)	0.572
Postoperative Pleurodesis			0.297
No	48 (100.0)	73 (94.8)	
Yes	0 (0.0)	4 (5.2)	
Recurrence			0.529
No	45 (93.8)	69 (89.6)	
Yes	3 (6.2)	8 (10.4)	
The interval between surgery and recurrence, days	262 [209, 316]	48.5 [30.0, 54.5]	0.036
Treatment after recurrence			1
Chest tube insertion	1 (33.3)	3 (37.5)	
Oxygen	1 (33.3)	2 (25.0)	
Surgery	1 (33.3)	3 (37.5)	

Data are presented as n (%), n/N (%), or median (interquartile range). ORC—oxidized regenerated cellulose; PGA—polyglycolic acid.

**Table 3:** Clinical characteristics of participants in individual studies.

			Number of Patients		Additional Sealant		Postoperative Pleurodesis ^¶^			Recurrence		
Author	Year	Age ^⸹^	ORC	PGA	ORC	PGA	ORC	PGA	*p*-Value	ORC	PGA	*p*-Value
Uramoto [13]	2014	38.2	8	177	Autologous blood	Fibrin sealant	0/8 (0.0)	14/177 (7.9)	0.408	0/8 (0.0)	23/177 (13.0)	0.276
Ozawa [8]	2018	21	57	55	No fibrin glue or autologous blood					13/57 (22.8)	2/55 (3.6)	<0.001
Iwazawa [14]	2021	20.9	37	238	No description					8/37 (21.6)	26/238 (10.9)	0.066

Data are presented as n (%) or n/N (%). ORC—oxidized regenerated cellulose; PGA—polyglycolic acid; SD—standard deviation. ⸹ median or mean age. ^¶^ pleurodesis after surgery due to prolonged air leakage.

## Data Availability

The data underlying this article will be shared by the corresponding author upon request.

## References

[1] Bobbio A., Dechartres A., Bouam S., Damotte D., Rabbat A., Régnard J.-F., Roche N., Alifano M. (2015). Epidemiology of spontaneous pneumothorax: Gender-related differences. Thorax.

[2] Gupta D., Hansell A., Nichols T., Duong T., Ayres J.G., Strachan D. (2000). Epidemiology of pneumothorax in England. Thorax.

[3] Kim D., Jung B., Jang B.-H., Chung S.-H., Lee Y.J., Ha I.-H. (2019). Epidemiology and medical service use for spontaneous pneumothorax: A 12-year study using nationwide cohort data in Korea. BMJ Open..

[4] Melton L.J., Hepper N.G., Offord K.P. (1979). Incidence of spontaneous pneumothorax in Olmsted County, Minnesota: 1950 to 1974. Am. Rev. Respir. Dis..

[5] Al-Tarshihi M.I. (2008). Comparison of the efficacy and safety of video-assisted thoracoscopic surgery with the open method for the treatment of primary pneumothorax in adults. Ann. Thorac. Med..

[6] Barker A., Maratos E.C., Edmonds L., Lim E. (2007). Recurrence rates of video-assisted thoracoscopic versus open surgery in the prevention of recurrent pneumothoraces: A systematic review of randomised and non-randomised trials. Lancet.

[7] Kim K.S. (2020). Polyglycolic acid sheet with fibrin glue technique without pleural abrasion in uniportal VATS for primary spontaneous pneumothorax. J. Thorac. Dis..

[8] Ozawa Y., Sakai M., Ichimura H. (2018). Covering the staple line with polyglycolic acid sheet versus oxidized regenerated cellulose mesh after thoracoscopic bullectomy for primary spontaneous pneumothorax. Gen. Thorac. Cardiovasc. Surg..

[9] Sakamoto K., Takei H., Nishii T., Maehara T., Omori T., Tajiri M., Imada T., Takanashi Y. (2004). Staple line coverage with absorbable mesh after thoracoscopic bullectomy for spontaneous pneumothorax. Surg. Endosc..

[10] Mukaida T., Andou A., Date H., Aoe M., Shimizu N. (1998). Thoracoscopic operation for secondary pneumothorax under local and epidural anesthesia in high-risk patients. Ann. Thorac. Surg..

[11] Oda R., Okuda K., Yamada T., Yukiue H., Fukai I., Kawano O., Matsui T., Tatematsu T., Yokota K., Nakanishi R. (2022). Comparison of the efficacy of novel two covering methods for spontaneous pneumothorax: A multi-institutional study. BMJ Open Respir. Res..

[12] Ebana H., Hayashi T., Mitani K., Kobayashi E., Kumasaka T., Mizobuchi T., Kurihara M., Takahashi F., Takahashi K., Seyama K. (2018). Oxidized regenerated cellulose induces pleural thickening in patients with pneumothorax: Possible involvement of the mesothelial–mesenchymal transition. Surg. Today.

[13] Uramoto H., Tanaka F. (2014). What is an appropriate material to use with a covering technique to prevent the recurrence of spontaneous pneumothorax?. J. Cardiothorac. Surg..

[14] Iwazawa T., Kadota Y., Takeuchi Y., Yokouchi H., Shiono H., Hayakawa M., Sakamaki Y., Kurokawa E., Nishioka K., Shintani Y. (2021). Efficacy of pleural coverage with polyglycolic acid sheet after bullectomy for postoperative recurrence of spontaneous pneumothorax in young patients: A multi-institutional cohort study. Gen. Thorac. Cardiovasc. Surg..

[15] Fujiwara T., Tanaka K., Toyoda T., Inage T., Sakairi Y., Ishibashi F., Suzuki H., Nakajima T., Yoshino I. (2020). Risk factors of postoperative recurrence of primary spontaneous pneumothorax. J. Thorac. Dis..

[16] Nakayama T., Takahashi Y., Uehara H., Matsutani N., Kawamura M. (2017). Outcome and risk factors of recurrence after thoracoscopic bullectomy in young adults with primary spontaneous pneumothorax. Surg. Today.

[17] Cattoni M., Rotolo N., Mastromarino M.G., Cardillo G., Nosotti M., Mendogni P., Rizzi A., Raveglia F., Siciliani A., Rendina E.A. (2020). Analysis of pneumothorax recurrence risk factors in 843 patients who underwent videothoracoscopy for primary spontaneous pneumothorax: Results of a multicentric study. Interact. Cardiovasc. Thorac. Surg..

[18] Asano H., Ohtsuka T., Noda Y., Kato D., Mori S., Nakada T., Matsudaira H. (2019). Risk factors for recurrence of primary spontaneous pneumothorax after thoracoscopic surgery. J. Thorac. Dis..

[19] Dagnegård H.H., Rosén A., Sartipy U., Bergman P. (2017). Recurrence rate after thoracoscopic surgery for primary spontaneous pneumothorax. Scand. Cardiovasc. J..

[20] Kutluk A.C., Kocaturk C.I., Akin H., Erdogan S., Bilen S., Karapinar K., Sezen C.B., Saydam O. (2018). Which is the Best Minimal Invasive Approach for the Treatment of Spontaneous Pneumothorax? Uniport, Two, or Three Ports: A Prospective Randomized Trail. Thorac. Cardiovasc. Surg..

[21] Mao Y., Zhang Z., Zeng W., Zhang W., Zhang J., You G., Wei Y. (2020). A clinical study of efficacy of polyglycolic acid patch in surgery for pneumothorax:a systematic review and meta-analysis. J. Cardiothorac. Surg..

[22] Porcel J.M., Lee P. (2021). Thoracoscopy for Spontaneous Pneumothorax. J. Clin. Med..

[23] Onuki T., Kawamura T., Kawabata S., Yamaoka M., Inagaki M. (2019). Neo-generation of neogenetic bullae after surgery for spontaneous pneumothorax in young adults: A prospective study. J. Cardiothorac. Surg..

[24] Woo W., Kim C.H., Kim B.J., Song S.H., Moon D.H., Kang D.-Y., Lee S. (2021). Early Postoperative Pneumothorax Might Not Be “True” Recurrence. J. Clin. Med..

[25] Yang H.C., Kim S., Yum S., Cho S., Kim K., Jheon S. (2017). Learning curve of single-incision thoracoscopic surgery for primary spontaneous pneumothorax. Surg. Endosc..

[26] Choi S.Y., Kim D.Y., Suh J.H., Yoon J.S., Jeong J.Y., Park C.B. (2018). New bullae formation in the staple line increases the risk of recurrent pneumothorax following video-assisted thoracoscopic surgery bullectomy for primary spontaneous pneumothorax. J. Thorac. Dis..

[27] Kurihara M., Mizobuchi T., Kataoka H., Sato T., Kumasaka T., Ebana H., Yamanaka S., Endo R., Miyahashira S., Shinya N. (2016). A Total Pleural Covering for Lymphangioleiomyomatosis Prevents Pneumothorax Recurrence. PLoS ONE.

[28] Biçer M., Bayram A.S., Gürbüz O., Senkaya I., Yerci O., Tok M., Anğ E., Moğol E.B., Saba D. (2008). Assessment of the efficacy of bio-absorbable oxidized regenerated cellulose for prevention of post-operative pericardial adhesion in the rabbit model. J. Int. Med. Res..

